# The pressure characteristics analysis of oil pulsation flow based on VMD

**DOI:** 10.1038/s41598-021-96860-0

**Published:** 2021-08-30

**Authors:** Ge Liu, Bin Chen

**Affiliations:** 1 School of Environmental Engineering, North China Institute of Science and Technology, Hebei, 065201 China; 2 School of Mechanical and Electrical, Hebei Key Laboratory of Safety Monitoring of Mining Equipment, North China Institute of Science and Technology, Hebei, 065201 China

**Keywords:** Engineering, Mathematics and computing

## Abstract

The pressure signal of oil pulsating flow is a kind of multi-component signal; in order to realise the effective separation of the multi-component pressure signal and extract its vibration characteristics, the pressure signal was decomposed by Variational Mode Decomposition (VMD). The slope criterion of the centre frequency is proposed to determine the number of components of VMD decomposition, and the method to judge the main components of the signal by energy value is proposed. The Hilbert envelope demodulation analysis was performed on the main components obtained. The results show that the proposed center frequency slope criterion method is effective in the VMD decomposition of the pressure signal of oil pulsating flow, which is used to decompose the pressure signal into 9 components. Four major components of the pressure signal are obtained by the correlation between each component and the pressure signal, and the energy value calculation of each component. The main component frequency of the pressure signal is one time, 6 times, 11 times and 14 times the frequency of the system spindle rotation; these are the sum of two cosine signals of close frequency and have the characteristic of beat vibration.

## Introduction

The turbulent pulsating flow of oil has been of nice interest to researchers for more than 50 years thanks to its intrinsic relationship with the physical nature of turbulence. From the review of Brereton and Mankbadi^1^, Gundogdu and Carpinlioglu's^2^ pulsating flow studies, it is often seen that the turbulent shear stress and viscous stress are derived from the uniform shear flow on the turbulent wall surface, according to the relative amplitude of these two stresses, the flow is split into different regions, specifically the laminar sublayer, the buffer layer and the turbulent outer layer. Thus, turbulent pulsating flow is additionally divided into the same region, and the flow velocity is often decomposed into the superposition of the time-uniform velocity and the periodic transient velocity. The turbulence transient velocity pulsation is that the random component of the transient local velocity determined by the average phase property.

The early studies of cyclic unstable turbulence in tubes chiefly specialise in the low pulsating amplitude and high frequency of rate of flow, and valuable results have been obtained within the proper resolution of scale parameters and the discussion of the interaction mechanism between shear wave and turbulence^3–6^. For example, Xuan et al.^7^ analysed the dynamics of Reynolds stresses and turbulent kinetic energy (TKE) in Langmuir turbulence exploitation knowledge of large-eddy simulations with the wave phase resolved. It’s found that the streamwise and spanwise Reynolds normal stresses and the Reynolds shear stress vary appreciably with the wave phase. The correlation impact will have an effect on the turbulence fluctuations at time scales around the wave period. Gerrard^8^ carried out experimental research on turbulent pulsating flow and employed photographic imaging technology to measure the local transient velocity of water flow. The flow includes a laminar fluidisation trend. Subsequently, Mizusgina et al.^9^ reported a comprehensive study of pulsating flow and obtained the phase mean velocity and turbulence intensity of the fluid from the information measured by an electrochemical method, which only covers 20 cycles for the difficulty concerned in the experiment, which limits the convergence of the results. McPherson et al.^10^ regarded as the momentum flux divergence of those internal waves suggest that just about 15% of the whole plume momentum are often transported out of the system by wave radiation, thus playing a vital role within the redistribution of momentum among the near-field plume.

At present, non-stationary signal analysis methods, such as the short-time Fourier transform (STFT), Hilbert Huang transform and analytical mode decomposition, have their different problems. The resolution of the time–frequency of STFT is restricted by the uncertainty principle, and Hilbert Huang transform has fuzzy time–frequency distribution such as endpoint impact and mode aliasing, analytical mode decomposition is simply suitable for analyzing stationary frequency multicomponent signals. For multicomponent time-varying, nonstationary signal analysis, Olhede and Walden^11^ put forward a form of the Generalised demodulation time–frequency analysis methodology applicable to many components such as amplitude modulation and frequency modulation signal, after nearly Zheng et al.^12^ proposes a new signal analysis methodology of generalized analytic modal decomposition (GAMD) to contain multiple time-varying modals, especially the non-stationary signal spectrum overlaps. However, the variational mode decomposition (VMD) may be a non-stationary signal decomposition method proposed in recent years, by transforming the signal decomposition problem into a variational constraint problem, the modal separation of multivariable signals is often realized. Researchers at home and abroad have obtained some important results. For example, Upadhyay and Pachori^13^ improved the ability of speech signal detection by exploiting VMD, believed that VMD with Wiener filter structure has higher robustness for extracting a noise signal, and VMD has higher accuracy for speech transient detection compared with the present methods. Li zhinong et al.^14^ proposed a mechanical fault diagnosing method that supported VMD, and compared with an ensemble empirical mode decomposition (EEMD) method, believed that VMD might effectively decompose the inherent mode of the signal, reveal the frequency structure of the rubbing fault knowledge, and distinguish the severity of the rubbing fault. Xiao huaishuo et al.^15^ obtained a group of stable modal components by VMD of the gas content sequence in detrend transformer flow, that provided a new idea for the prediction model in different areas of the power system. The VMD transforms the problem of solving the modal bandwidth into a constrained optimization problem and decomposes every mode around a certain central frequency, which may adaptively understand the frequency domain segmentation of the signal and the effective separation of every component.

The variational mode decomposition due to need to verify the parameters such as number of penalty function and component decomposition in step with the previous information and the characteristics of the signal, and there is human drawback as subjective choice in signal analysis, several researchers put forward many improving methods, such as a generalized variational mode decomposition method is advocate supported the influence of the setting of penalty factors and the number of components on the filtering characteristics of VMD method^16^, an automatic parameter search strategy based on the influence of Feature factor of Envelope Spectrum (EFF) was proposed^17^; the particle swarm optimization algorithm for optimal impact parameter combination of the variable-modal decomposition algorithm is employed to line the penalty parameters and number of components of the variable-modal decomposition algorithm^18^; the parameter optimized variational mode decomposition (POVMD) relies on the parameter optimized of variational mode decomposition^19^, etc.

According to the multi-scale and quasi-periodic non-stationary signal characteristics of the oil pulsating flow, the VMD analysis of the oil pressure fluctuation signal was carried out, and a way was proposed to determine the quantity of VMD decomposed components by exploitating the slope criterion of the center frequency, similarly the energy value to evaluate the main components of the signal. Therefore, the outline of the rest of this paper is as follows. In “Experimental devices and methods” section, the oil pulsation flow tested experimental equipment set up. In “Validation of VMD analysis method” section, the slope criterion of the center frequency was put forward and validated its effectiveness. The intrinsic mode function (IMF) is decomposed by VMD to get some IMF components, Then the Hilbert transformation is employed to estimate the signal envelope of every main component to obtain the frequency component information of every main component in “Results and analysis” section. Finally, we tend to conclude in “Conclusions” section.

## Experimental devices and methods

### Experimental device

**Figure 1:**
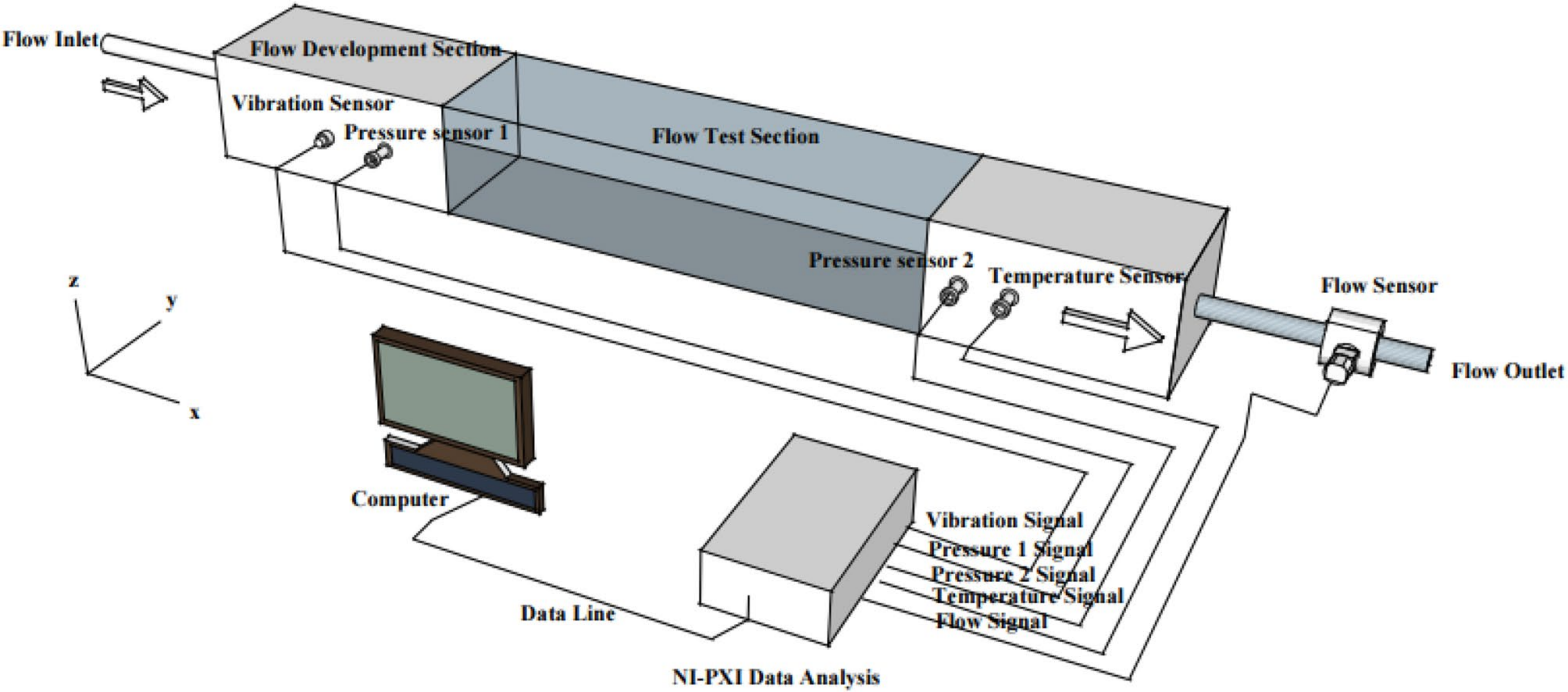
The schematic diagram of the experimental device of oil pulsation flow.

The schematic diagram of the experimental device of oil pulsation flow is shown in Fig. 1, which mainly includes two parts: the experimental pipeline and the information acquisition system. The section of the experimental pipe is 0.04 × 0.04 m, square tube with a length of 0.5 m, and there is a circular inlet and outlet with a diameter of 24.00 mm at each end, divided into two parts: the development section and the test section. In order to observe the movement of oil, the top and front of the test section are fabricated from transparent glass, whereas the bottom and rear walls are fabricated from steel plates. The cartesian coordinate system is established as shown in Fig. 1; The origin is the vertex at the bottom left of the experimental pipeline, the x-direction is the flow direction of oil, the y-direction is the spanwise direction, and the z-direction is the normal direction.

The data acquisition system mainly includes oil pressure transmitter, pipeline vibration transmitter, temperature transmitter and oil flow transmitter, NI-PXI data acquisition card and LabVIEW data analysis and processing software. The pressure transmitter adopts YP-01s with a measuring range of  − 0.1–2.0 MPa, the accuracy of 0.2% and output of 0-5VDC, which’s accustomed to collect pressure modification signals of the oil at the inlet and outlet of the experimental pipeline. The vibration transmitter set at the entrance of the experimental pipeline adopts LC0123 with an accuracy of 0.08% to notice the vibration signal of the experimental pipeline. NI-PXI data acquisition card employed is the NI company production of NI PXI - 6133 acquisition card, the acquisition card is a synchronous sampling multi-function data acquisition equipment, will provide 8 analog input channels (14 bits, the rate of every channel is as high as 2.50 MS/s), article 8 digital I/O lines, two 24 bits counter and trigger, mainly converts electrical signals to digital signals, then transmit to the LabVIEW software in a computer, the pressure signal is collected real-time display and storage. The temperature adopts a TK52D transmitter; the preciseness is 0.2%FS, the range is 0–200℃, the output is 0–5VDC. The flow rate adopts FR80 type transmitter; the accuracy is ± 0.2%, output 4–20 mA. The data of temperature transmitter and flow transmitter are employed as a reference for the analysis of oil pulsation characteristics. Real-time data such as pressure and vibration collected by the system were transmitted to LabVIEW software in the computer through NI-PXI acquisition board, and the dynamic parameter information of oil pressure and acceleration were filtered, denoised and analyzed.

The oil employed in the experiment was 25# transformer oil, and the oil sample was obtained by filtering the transformer oil with neutral filter paper. The oil sample is placed in the oil tank and fed into the experimental pipeline through a peristaltic pump, the inlet velocity is V_0_ = 0.0362 m/s. The pressure sensors 1 and 2 are respectively installed on both sides of the test section, with 150 mm and 350 mm distance from the inlet end respectively. The Reynolds number of the oil in the test section Re = V_0_*D/ν, where ν is the kinematic viscosity of the oil, D is the equivalent diameter of the test section, which is 0.0497 m, thus Re = 138.4. After the oil passes through the pipeline development section, the flow enters the test section and enters the oil tank through the tail section, forming a cycle. The oil was placed into the experimental pipeline for 10 min, after the oil index in the system modified steady, the data sampling of every parameter was started.

### Experimental methods

LabVIEW software is employed to compile the front panel and program block diagram of data acquisition. The front panel mainly contains the parameter setting area and the waveform displayed area, with the button controls such as filter, data saving and image saving; the function of filter beginning, experimental data and image storage is commonly realised. The displayed area of the filter diagram is to display the changes of the sample values of inlet pressure, outlet pressure, flow rate, temperature and vibration (acceleration) of pipeline oil with time in real-time. The parameter setting area selects parameters such as connection mode, sampling rate, sampling number and sampling method of the NI-DAQmx task signal. The choice of specific parameters is shown in Table 1.

**Table 1:** The selection of specific parameters.

Name	Connection mode	Sampling rate	Sampling number	Sampling method
Pressure, flow rate, temperature channal	RSE	1000	6000	Continuous samplies
Vibration channal	Pseudo differential	1000	6000	Continuous samplies

The program block diagram corresponding to the front panel, will appear in the front panel input and output program with some (cycle, sequence etc.) structure with the line segment connection, in order that the front panel controls will communicate with each other.

### The VMD analysis method

#### The VMD method

The VMD decomposes the signal into several inherent modal functions (IMF), that is an adaptive non-recursive signal decomposition method. An interactive direction multiplier algorithm is employed to solve the process based on the constrained variational optimisation problem. The estimation steps of every component's bandwidth are as follows,

- Transform the real signal into an analytical signal by Hilbert, to obtain the unilateral spectrum of the signal;
- To modulate the spectrum of the central frequencies of every component into the base frequency band;
- The bandwidth of the component is estimated by H^1^ gaussian smooth demodulation signal.

Then the constraint variational problem model of VMD is,

1 $$ \mathop {\min }\limits_{{\left\{ {y_{k} } \right\}\left\{ {\omega_{k} } \right\}}} \left\{ {\sum\limits_{k = 1}^{K} {\left\| {\partial_{t} \left[ {\left( {\delta \left( t \right) + \frac{j}{\pi t}} \right) \cdot y_{k} \left( t \right)} \right]e^{{ - j\omega_{k} t}} } \right\|_{2}^{2} } } \right\}\quad s.t{.}\sum\limits_{k = 1}^{K} {y_{k} \left( t \right)} = y\left( t \right) $$

where K is the number of components decomposed by VMD and $y_{k} \left( t \right)$ is the k-order component of the signal $y\left( t \right)$, $\left\{ {y_{k} } \right\},\;\left\{ {\omega_{k} } \right\}$ respectively representing every component and its central frequency after VMD decomposition.

The constrained optimisation problem of Eq. (1) is commonly transformed into a non-constrained optimisation problem by applying the quadratic penalty parameter and the Lagrangian multiplier, and the equation adjusted by the Lagrangian multiplier is,

2 $$ \begin{aligned} & L\left( {\left\{ {y_{k} } \right\},\left\{ {\omega_{k} } \right\},\lambda } \right) = \\ & \alpha \sum\limits_{k = 1}^{K} {\left\| {\partial_{t} \left[ {\left( {\delta \left( t \right) + \frac{j}{\pi t}} \right) \cdot y_{k} \left( t \right)} \right]e^{{ - j\omega_{k} t}} } \right\|_{2}^{2} } + \left\| {y\left( t \right) - \sum\limits_{k = 1}^{K} {y_{k} \left( t \right)} } \right\|_{2}^{2} + \left\langle {\lambda \left( t \right),y\left( t \right) - \sum\limits_{k = 1}^{K} {y_{k} \left( t \right)} } \right\rangle \\ \end{aligned} $$

where $\alpha$ is the quadratic penalty parameter, and $\lambda \left( t \right)$ is the Lagrangian multiplier.

Equation (2) is solved by using the interactive direction multiplier algorithm, and the estimated value of every component in the frequency domain is,

3 $$ \hat{Y}_{k}^{n + 1} \left( \omega \right) = \frac{{\hat{Y}\left( \omega \right) - \sum\limits_{i \ne k} {\hat{Y}_{i} \left( \omega \right) + {{\hat{\lambda }\left( \omega \right)} \mathord{\left/ {\vphantom {{\hat{\lambda }\left( \omega \right)} 2}} \right. \kern-\nulldelimiterspace} 2}} }}{{1 + 2\alpha \left( {\omega - \omega_{k} } \right)^{2} }} $$

where $\hat{Y}\left( \omega \right)$,$\hat{Y}_{i} \left( \omega \right)$, $\hat{\lambda }\left( \omega \right)$ and $\hat{Y}_{k}^{n + 1} \left( \omega \right)$ respectively represent the Fourier transform of $y\left( t \right)$,$y_{i} \left( t \right)$,$\lambda \left( t \right)$, and $y_{k}^{n + 1} \left( t \right)$.

Equation (3) contains the Wiener filter structure, and the time-domain expression of every component is obtained by the real part of Fourier transform of the filtering signal.

The updated expression of the center frequency of every component is,

4 $$ \omega_{k}^{n + 1} = \frac{{\int_{0}^{\infty } {\omega \left| {\hat{Y}_{k} \left( \omega \right)} \right|^{2} d\omega } }}{{\int_{0}^{\infty } {\left| {\hat{Y}_{k} \left( \omega \right)} \right|^{2} d\omega } }} $$

#### Determination method of VMD component quantity

Since the number K of decomposition components has to be determined firstly in the decomposition process of VMD, different signal characteristics are different, and the number of components of VMD decomposition is not the same. Consistent with the slope of the center frequency of every component of VMD decomposition, a slope criterion of the center frequency is proposed to determine the number of components of VMD decomposition. After every component decomposed by VMD is transformed by Hilbert, Hilbert envelope demodulation is to calculate the Hilbert transform of the original signal x(t) containing modulation information and get the imaginary part x '(t) of the original signal. The envelope of the original signal is P(t) = (x(t)^2^ + x′(t)^2^)^1/2^ from x(t) and x′(t). Then the envelope demodulation spectrum of the original signal is obtained by the spectrum analysis of the envelope signal. It is one of the common methods to analyse AM and FM signals, the specific principle is detailed in the literature^20^. The signal to be analysed is expressed as,

5 $$ y\left( t \right) = {\text{Re}} \left[ {\sum\limits_{k = 1}^{K} {a_{k} \left( t \right)e^{{j\theta_{k} \left( t \right)}} } } \right] $$

where $a_{k} \left( t \right)$ represents the instantaneous amplitude of every component, and $\theta_{k} \left( t \right)$ represents the instantaneous phase angle of every component. Then the instantaneous frequency of every component is,

6 $$ f_{k} \left( t \right) = \frac{{d\theta_{k} \left( t \right)}}{2\pi dt} $$

The criterion method is as follows: first, it is assumed that the center frequency of every component of VMD decomposition of K-component signal iterates in the order from small to large, and their slope will increase with the increase of the number of components; Then the slope of the instantaneous mean frequency of every component is consistent with the trend of the modification of the central frequency, and the number of decomposition continues to increase; If it is inconsistent, it is seemingly that VMD has been over decomposed, and the number of VMD is determined by this criterion. Specific implementation steps are as follows,

- Initialize the decomposition quantity K of VMD, K = 1;
- K = K + 1; implement the iterative decomposition process of VMD;
- Hilbert transformation was performed on every component after decomposition, and the center frequency $f_{zk} \left( t \right) = {{\omega_{k} \left( t \right)} \mathord{\left/ {\vphantom {{\omega_{k} \left( t \right)} {2\pi }}} \right. \kern-\nulldelimiterspace} {2\pi }}$ and instantaneous mean frequency $\overline{f}_{k} \left( t \right)$ of every component were obtained;
- Compare the slope of the center frequency and the mean value of instantaneous frequency of every component, and repeat (b)–(d) if it is in keeping with the symbol $\frac{{d\overline{f}_{k} \left( t \right)}}{dt}$ and $\frac{{df_{zk} \left( t \right)}}{dt}$; Otherwise, finish the VMD program, and K = k − 1; you get the number K of components of the VMD decomposition.

## Validation of VMD analysis method

**Figure 2:**
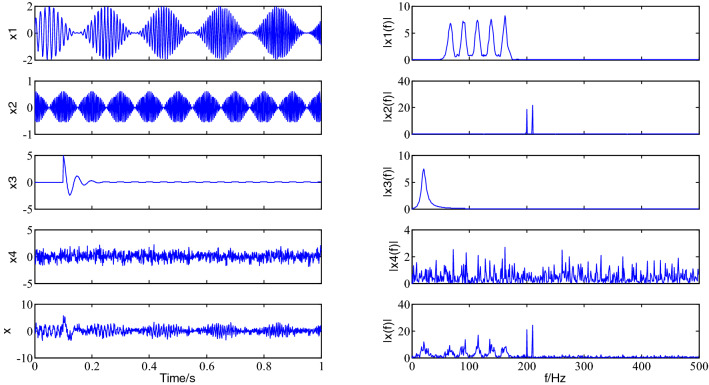
The time-domain and frequency-domain diagrams of every component.

To validate the effectiveness of the pressure feature extraction of the vibration signal of the projected VMD, a typical characteristic of multicomponent simulation signal x (t) is investigated, that it contains the amplitude modulation (AM) and frequency modulation (FM) of the time-varying signal ×1 (t), a clap-frequency vibration signal ×2 (t), the impact of the exponential decay signals ×3 (t) and noise analogue signal ×4 (t), its mathematical expression is,

7 $$ \left\{ \begin{gathered} x1\left( t \right) = \left[ {1 + \sin \left( {2\pi 5t} \right)} \right] \cdot \sin \left( {2\pi 60t + 2\pi 60t^{2} } \right) \hfill \\ x2\left( t \right) = 0.3\cos \left( {2\pi 200t} \right) + 0.3\cos \left( {2\pi 210t} \right) \hfill \\ x3\left( t \right) = 100e^{ - 30t} \cos \left( {2\pi 20t} \right) \, t > 0.1 \hfill \\ x4\left( t \right) = 0.7randn(1,N) \hfill \\ x\left( t \right) = x1\left( t \right) + x2\left( t \right) + x3\left( t \right) + x4\left( t \right) \hfill \\ \end{gathered} \right. $$

where N is the data length. The signal x (t) is sampled for 1 s at a sampling frequency of 1000 Hz, and the time-domain and frequency-domain diagrams of every component are drawn, as shown in Fig. 2.

The slope criterion of the center frequency is employed to determine the number K of components of VMD decomposition, the center frequency and the mean values of instantaneous frequency of every component after VMD decomposition for 14 times are shown in Table 2. When K = 14, the average instantaneous frequency of the 14th component of pressure signal decomposition is 413.9253 Hz, which is less than the instantaneous mean frequency of the component corresponding to K = 13, 430.9767 Hz; at the same time, the center frequency of the VMD decomposition component is 450.2397 Hz and 482.9588 Hz respectively at K is 13 and 14. This is, the slope of the instantaneous mean frequency changed compared to the slope of the center frequency of the component of VMD decomposition when K is 14, it indicates that if the VMD is decomposed to the 13th time and further decomposed, the phase distinction of components will be adjusted in the opposite direction (downward trend) from the upward trend of the previous decomposition, the data shows that the instantaneous mean frequency decreases, that is, the over-decomposition happens, that the number K of components of the simulation signal of VMD decomposition is 13. Therefore, it is possible to use the slope criterion of center frequency to determine the number K of component of VMD decomposition.

**Table 2:** The center frequency and the mean values of instantaneous frequency of every component.

	1	2	3	4	5	6	7	8	9	10	11	12	13	14
f_zk_	21.6214	65.7379	89.5505	114.6743	138.2358	162.3067	204.6448	255.1097	291.1580	329.3023	372.8922	416.6536	450.2397	482.9588
f_k_	20.9825	65.8180	89.8295	118.8020	135.7863	164.7838	201.9950	254.8936	289.9943	327.9909	370.9821	413.9947	430.9766	413.9253

**Figure 3:**
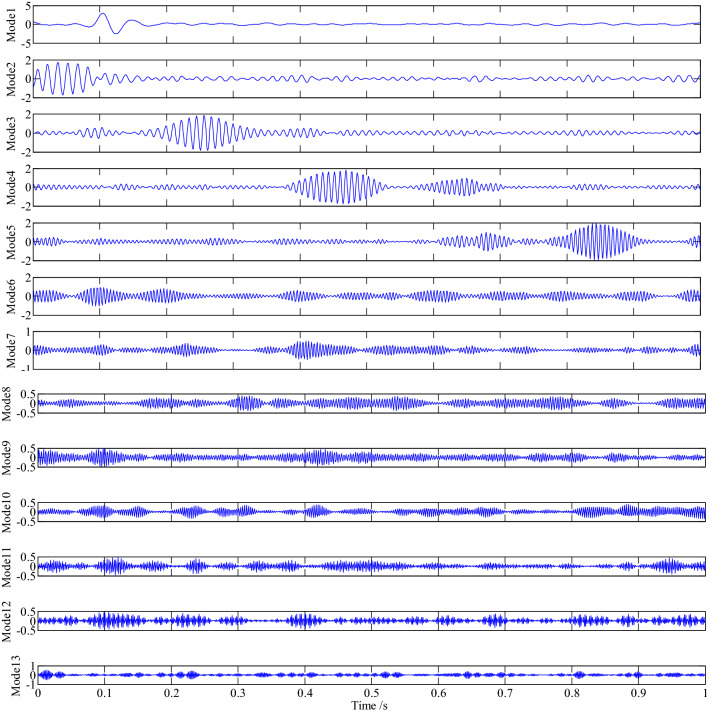
The time domain waveform of the 13 components.

The VMD decomposition was carried out on the simulation signal, and the time domain waveform of the 13 components obtained was shown in Fig. 3.

As is often seen from Fig. 3, every component of the simulation signal is well decomposed; among them, mode1 is the impact signal ×3(t) of the simulation signal, a large impact amplitude occurs at 0.1 s, and then attenuation is carried out according to exponential law. The mode2-5 is the decomposition component of the AM and FM signal ×1(t) of the simulation signal. It is often seen that with the extension of time, the main frequency of the signal increases gradually, and there is an amplitude modulation phenomenon of beat vibration; It is often seen from mode4–5 that a wave packet with a small amplitude appears in the interval of 0.6–0.7 s during the sampling time, that is consistent with the five main frequencies in every component of VMD decomposition in the signal expression of ×1(t). Mode6 is the decomposed component of the simulation signal ×2(t); that is, mode6 is the beat-vibration amplitude modulation phenomenon formed by cosine signals with frequencies of 200 Hz and 210 Hz; The mode7–13 is the noise component ×4(t) of the simulation signal.

By comparing the energy values of the 13 components of VMD decomposition, it was found that the energy values of the first 6 components were all larger than the average energy value of 82.9714 of the simulation signal (as shown in Table 3). In addition, the correlation between the decomposed the previous 6 components and the simulated signal is obvious; Then the central frequency slope criterion is employed to determine the number of components of VMD decomposition, adding the judgment of component energy value and the true component of the signal is often obtained.

**Table 3:** The energy value and correlation of every component.

	1	2	3	4	5	6	7	8	9	10	11	12	13
Energy value	209.4777	123.9044	148.9425	166.1096	167.7486	99.8151	18.9376	24.5480	25.0651	20.7660	21.6171	25.2069	26.4891
Correlation	0.4173	0.3620	0.3955	0.4571	0.4419	0.3208	0.2051	0.1953	0.1910	0.1845	0.1824	0.1949	0.1852

**Figure 4:**
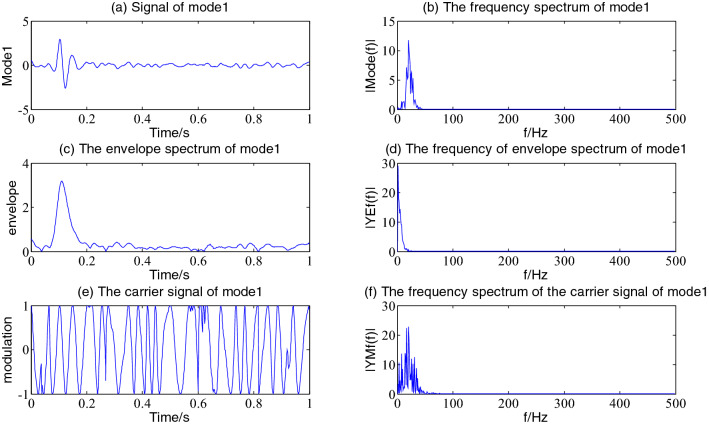
The envelope demodulation distribution diagram of mode1.

**Figure 5:**
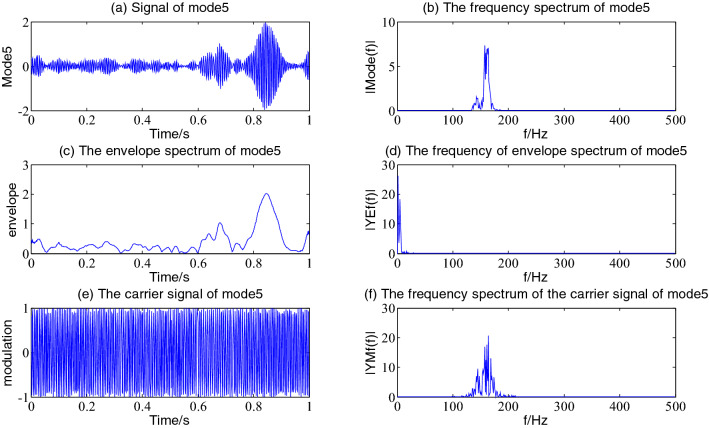
The envelope demodulation distribution diagram of mode5.

**Figure 6:**
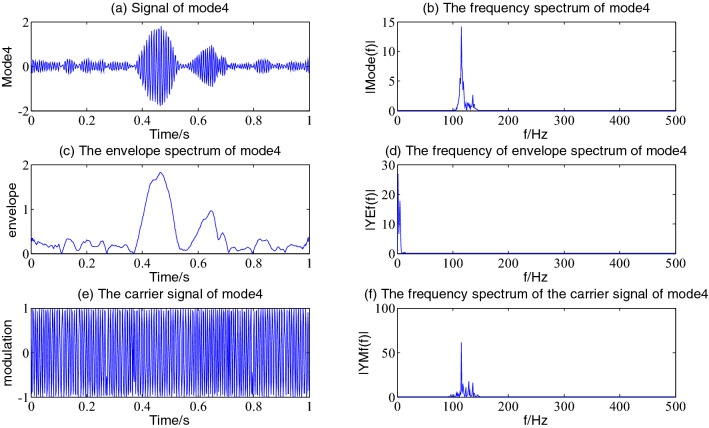
The envelope demodulation distribution diagram of mode4.

**Figure 7:**
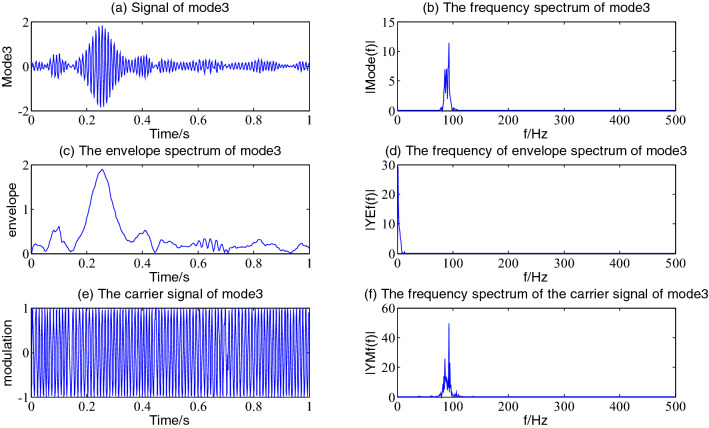
The envelope demodulation distribution diagram of mode3.

**Figure 8:**
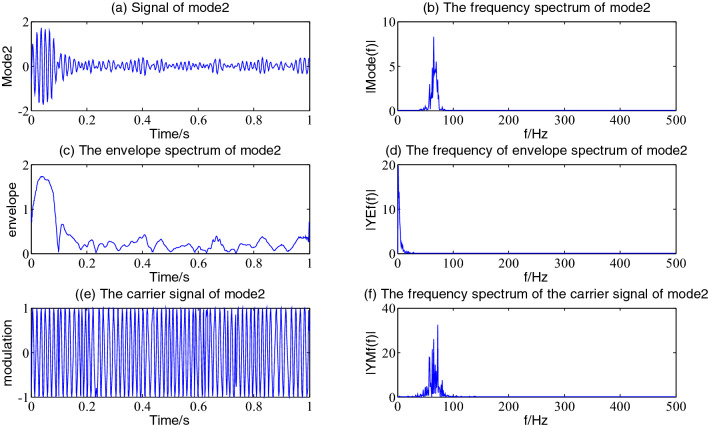
The envelope demodulation distribution diagram of mode2.

**Figure 9:**
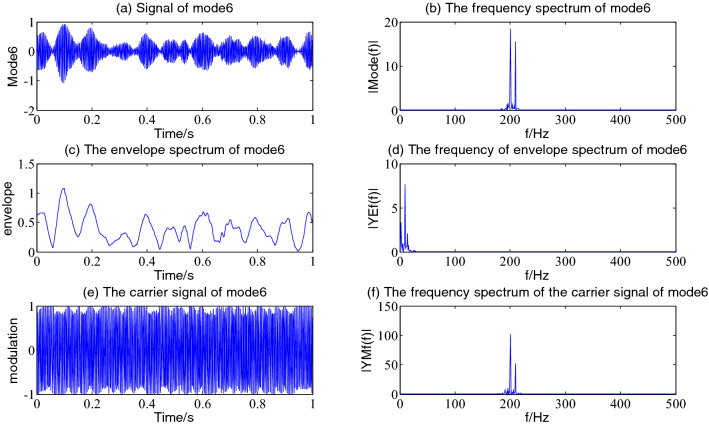
The envelope demodulation distribution diagram of mode6.

The Hilbert envelope demodulation analysis is performed on the first 6 components of the simulation signal decomposed by VMD, namely the first, fifth, fourth, third, second and sixth components with large energy values, as shown in Figs.4, 5, 6, 7, 8 and 9, more detailed information of every component is often obtained.

It is often seen from Fig. 4 that the first component of the simulation signal after VMD decomposition represents the information of the exponentially attenuated impact signal. Figure 4a shows that the occurrence time of the impact signal is 0.1 s, in the spectrum diagram in Fig. 4b, the dominant frequency is 20.51 Hz, the envelope spectrum and its spectrum after Hilbert envelope demodulation are shown in Fig. 4c and d. The envelope of mode1 contains the noise of other components, however the amplitude of impact information dominates. Figure 4e and f show the decomposed carrier signal and its spectrum of mode1; it is often seen that its carrier signal is an FM signal with the main frequency of 20.51 Hz.

Figures 5, 6, 7 and 8 shows the envelope demodulation information of the 2nd—5th component, the decomposed component of the AM and FM signal ×1(t). It is often seen from Figs. 5, 6, 7 and 8a that the amplitude of every component is different at different moments, and the distribution will increase successively along mode2-5, and the frequency spectrum of the different components also will increase in sequence in Figs. 5, 6, 7 and 8b.

Figure 9 is the reflection of the beat vibration signal ×2(t). It is often seen from Fig. 9a that the beat vibration phenomenon of the signal is extremely obvious, Fig. 9b and f show that the main frequencies of mode6 and its demodulated carrier signal are both 200 Hz and 210 Hz, Fig. 9c and d show a frequency of the envelope waveform and its spectrum is 10 Hz.

**Figure 10:**
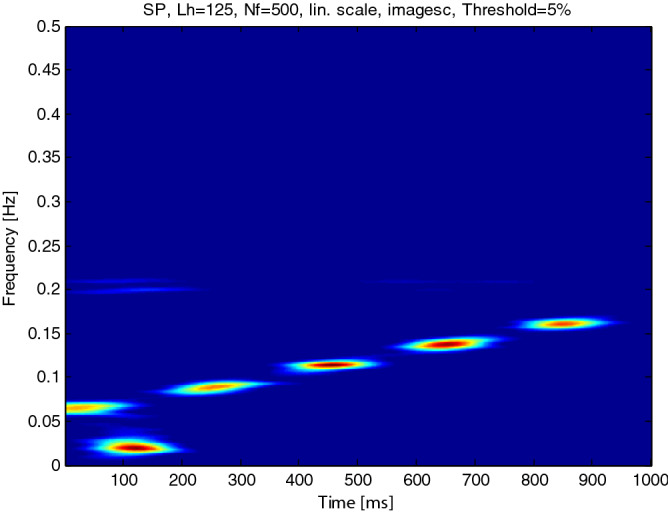
The time–frequency diagram of every component.

Figure 10 shows the time–frequency diagram of every component of the simulation signal after VMD decomposition, it is often seen that the maximum amplitude of the impact signal with a frequency of 20 Hz occurs at 0.1 s, and the amplitude of the AM and FM signal is different at different moments. However, the amplitude of the beat signal is small in the time–frequency diagram because of its small amplitude. It is often seen that the true component of the signal is often obtained by using the slope criterion of the center frequency to determine the number of components decomposed by VMD combined with the judgment of energy value.

## Results and analysis

According to the experimental device of oil pulsating flow, as shown in Fig. 1, the cross-section of the experimental pipeline is 0.04 × 0.04 m, the length is 0.5 m square, and there is a round inlet and outlet with a diameter of 24.00 mm at both ends, which shows that the inlet of the experimental pipeline is a sudden expansion section, and the input oil is powered by a transmission pump with a rotating shaft frequency 30 Hz of the system. The inlet velocity of the experimental apparatus is therefore pulsating. Latonell and Pollard^21^ showed that the onset of fluid instability depends on the inlet velocity. More generally, the upstream flow conditions, i.e., the velocity distribution in the inflow section and the intensity of residual turbulence, are the noise sources that trigger flow oscillations. According to the Re = 138.4 of the experimental segment, at this low Reynolds number, the steady flow in the experimental segment shows that the flow downstream of the contraction section is asymmetrical. The jet from the abrupt expansion deflects toward the wall, resulting in the formation of a unilateral recirculation region. This asymmetry is caused by the Komenda-type wall attachment already observed in a sudden expanding flow in a plane of symmetry.

When the Reynolds number increases above the critical value, the separation surface cannot remain attached, and the unsteady flow state begins. The low-frequency axial oscillation of the reattachment point and the slow, eddy motion of the jet was observed. This phenomenon is associated with the periodic discharge of unstable recirculation zones, resulting in alternating laminar and turbulent flows. The resulting flow is non-stationary and intermittent^22^.

It was further shown that in the absence of a finite disturbance, the geometric complexity of the conduit resulted in an adverse pressure gradient and jet deflection closer to the wall, resulting in a flow transition^23^. Moreover, the beginning of the transition depends on the location and time of the fluid. Therefore, to obtain the multi-scale and quasi-periodic non-stationary signal characteristics of the pulsating flow of oil, oil was put into the experimental pipeline for 10 min. After the change of various indicators of the oil in the system was relatively stable, data sampling of various parameters was started. The center frequency slope criterion proposed in  “Validation of VMD analysis method” section is used to determine the number of components of VMD decomposition, and the method of energy value to judge the main components of the signal is used to analyse and process the oil pressure fluctuation signal. The VMD is used to decompose the pressure vibration signal by IMF, and the envelope is decomposed by Hilbert transform to obtain the frequency component information of each major component.

### VMD decomposition of pressure signal

**Figure 11:**
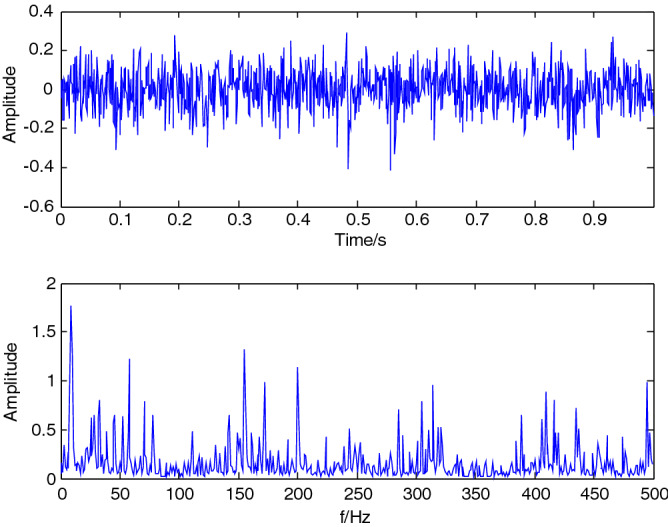
The time-domain distribution and spectrum of the oil pressure signal.

The time-domain distribution and frequency spectrum of the pressure signal 2 (x/D = 7, Re = 138.4) of oil pulsating flow are shown in Fig. 11; the oscillation period was 0.15D/V_0_(dimensional time was 0.21 s). This oscillation begins with the growth of the recirculation zone, followed by the movement of vortices around the pipe axis. Asymmetric separation of the relative parts of the cross-section was also observed. It is often seen that the pressure signal may be a collection of multi-component signals containing pulsating impact components, it is troublesome to distinguish the amplitude and occurrence time of those components from the time-domain distribution; Some frequency values with massive amplitude is usually obtained in the frequency domain graph, such as 8.789 Hz, 57.62 Hz, 155.3 Hz, 200.2 Hz, 314.5 Hz and 409.2 Hz, etc. however, their composition is relatively complicated, and it is troublesome to conduct in-depth analysis.

**Figure 12:**
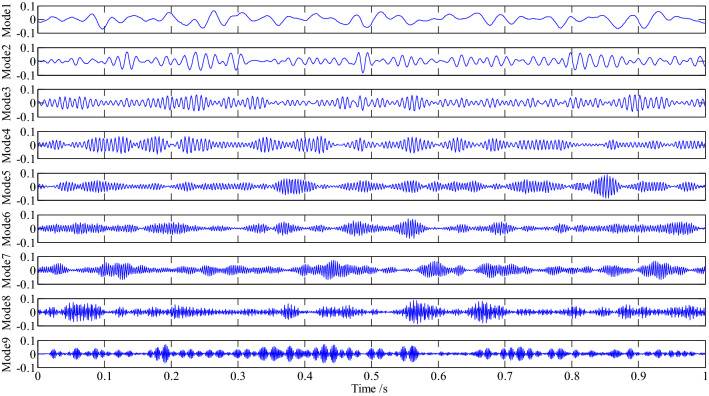
The nine mode components of VMD decomposition.

VMD decomposition was performed on the pressure signal, and the slope criterion of center frequency was employed to determine the number of components of VMD decomposition; the nine mode components were obtained as shown in Fig. 12.

The energy value, the central frequency corresponding to every component and its correlation with the pressure signal are shown in Table 4. The main components are found by comparing the energy value of every component with the mean energy value. The mean energy value of the calculated pressure signal is 0.5849, and the components greater than the mean value are the first, eighth, fourth and seventh components. It is often seen from the correlation between every component and the pressure signal that the filtered component has a more obvious correlation.

**Table 4:** The energy value and correlation of every component.

	1	2	3	4	5	6	7	8	9
Central frequency	22.6786	66.8973	130.4314	173.7091	235.4988	279.9022	320.1375	408.5228	466.2466
Energy value	0.7219	0.5740	0.5123	0.6080	0.5320	0.4418	0.5963	0.7045	0.5738
Correlation	0.3669	0.3615	0.3620	0.2831	0.3605	0.3381	0.3601	0.3881	0.3541

It is often seen from Fig. 12 that mode1, the main component of pressure signal decomposed by VMD, may be a periodic amplitude modulation signal that represents the fundamental development trend of pressure signal; The mode4, mode7 and mode8 are the beat vibration signals of various frequencies respectively; these four components are the main components of the pressure signal; the mode2, mode3, mode5, mode6 and mode9 are the transition components between the main components, that occupy a small amount of energy. It is often seen that the amplitude distribution of every component is relatively obvious during the sampling time (Fig. 12).However the bandwidth of every component is still large, so the envelope demodulation analysis is carried out on them.

### Envelope demodulation distribution of the main component

**Figure 13:**
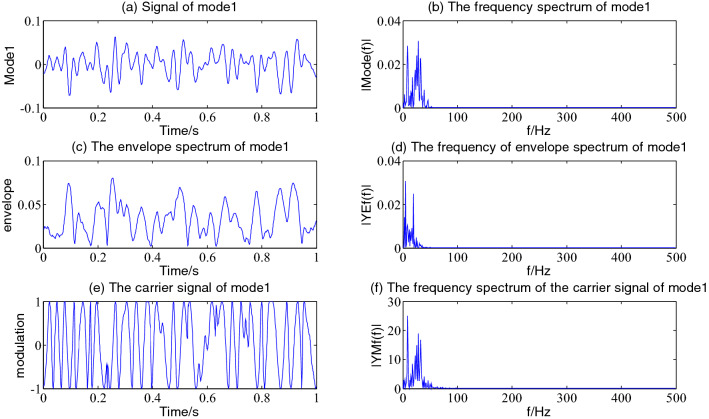
The envelope demodulation distribution diagram of mode1.

**Figure 14:**
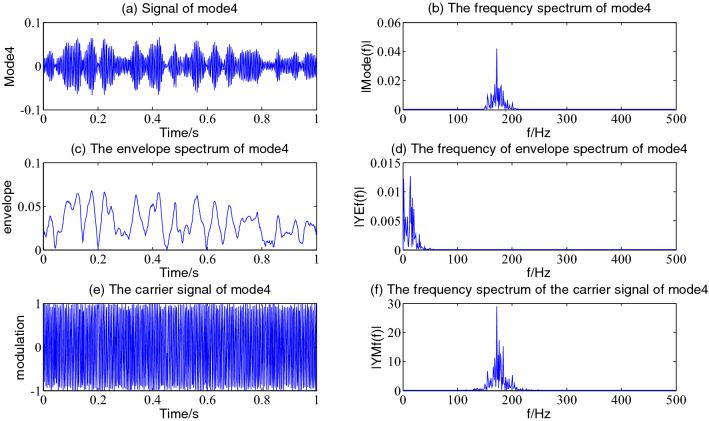
The envelope demodulation distribution diagram of mode4.

**Figure 15:**
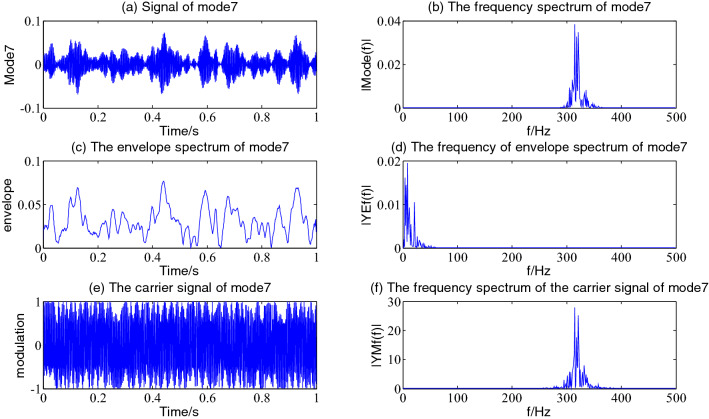
The envelope demodulation distribution diagram of mode7.

**Figure 16:**
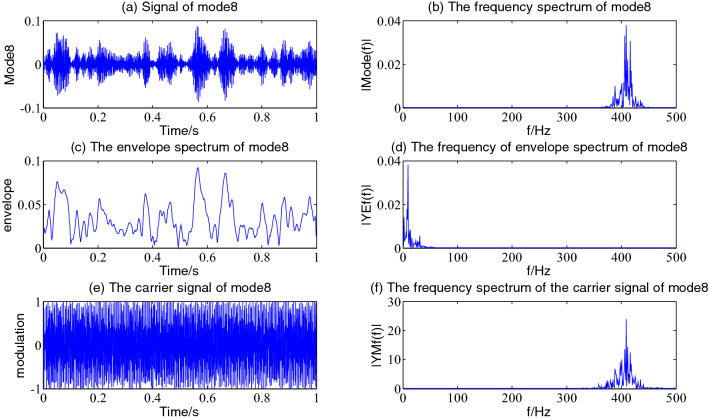
The envelope demodulation distribution diagram of mode8.

Figures 13, 14, 15 and 16 shows the envelope demodulation distribution diagram of the main components of the pressure signal after VMD decomposition. As is often seen from Fig. 13, the frequency spectrum of mode1 (Fig. 13a) has two main frequencies of 8.79 Hz and 28.32 Hz, and the frequency of 28.32 Hz is close to the shaft frequency of the system's transmission pump, 30 Hz. The frequency of envelope demodulation is 4.88 Hz and 19.53 Hz (Fig. 13d); thus, mode1 results from amplitude modulation of the rotating frequency of a spindle through a 19.53 Hz modulation signal. In Fig. 13f, it is often seen that the carrier signal after the envelope demodulation of mode1 is accompanied by a side frequency with an interval of 4.88 Hz on both sides of the frequency of 28.32 Hz, so the carrier signal is an FM signal. Moreover,19.53 Hz = 28.32 Hz-8.79 Hz = 2 × 2 × 4.88 Hz, namely, the mode1 component of the pressure signal indicates that the pressure signal is the result of amplitude modulation of a central frequency (28.32 Hz + 8.79 Hz)/2 = 18.65 Hz carrier being modulated by a frequency of 2 × 4.88 Hz. The pressure signal is, therefore an AM and FM signal.

Figure 14 shows the envelope demodulation distribution of mode4. It is often seen from Fig. 14a that mode4 has the characteristics of FM; the envelope frequency is 13.67 Hz through Hilbert envelope demodulation (Fig. 14d). It is often seen in Fig. 14b and f that the main frequency of mode4 and its carrier spectrum is 171.9 Hz, that is 6 times the main frequency of mode1 (28.32 Hz); there are several edge frequencies distributed on both sides, among that the amplitude of frequency in the frequency band from 158.23 to 185.57 Hz is large, that is determined by the characteristics of the FM. That is, the sum of the two cosine signals with the main frequency of 158.23 Hz and 185.57 Hz, respectively, and the result is the product of the cosine of half of the subtraction between the two main frequencies and the sine of half of the sum of the two main frequencies; the main frequency of mode4 is 171.9 Hz (= (158.23 + 185.57) /2 Hz) and the main frequency of the envelope is 13.67 Hz (= (185.57–158.23)/2 Hz).

Figure 15 shows the envelope demodulation distribution of mode7. From Fig. 15b, it is often seen that the spectrum of mode7 contains two main frequencies of 314.5 Hz (11 times of mode1's main frequency of 28.32 Hz) and 335 Hz; The frequencies after the Hilbert envelope demodulation are 3.906 Hz, 8.789 Hz and 21.48 Hz, respectively, with high amplitude; Moreover, it is often seen from the carrier signal waveform in Fig. 15e that the carrier signal after the Hilbert envelope demodulation still has the amplitude modulation feature. Thus, there is two main frequency of 314.5 Hz and 335 Hz component after mode7 further decomposed, the envelope frequency of two components is 3.906 Hz, 4.883 Hz, respectively. It is often seen that the 8.789 Hz of the envelope spectrum frequency in Fig. 15 is the sum of 3.906 Hz and 4.883 Hz, while the 21.48 Hz is about the difference between 314.5 Hz and 335 Hz, which is the characteristic of the beat vibration of two signals with close frequencies.

Figure 16 shows the distribution of the Hilbert envelope demodulation of mode8. It is often seen that in Fig. 16b, there are 388.7 Hz, 416 Hz, and 425.8 Hz frequency values with large amplitude on both sides of the main frequency 398.9 Hz,409.2 Hz, their intervals are 9.766 Hz, shown that mode8 is the result of beat vibration of two dominant frequencies as shown in Fig. 16f, the side frequency 398.9 Hz of the carrier signal spectrum is 14 times of the main frequency 28.32 Hz of mode1. It is often seen from Fig. 16d that 9.766 Hz is the main frequency of the envelope, these 398.9 Hz,409.2 Hz two main frequency signals are modulated by 9.766 Hz signal; The mode8 component is an FM and beat vibration signal.

It can be seen that the time domain distribution and spectrum of oil pulsating flow pressure signal 2 (at x/D = 7, Re = 138.4) are determined by the slope criterion of central frequency and the number of 9 components of VMD decomposition is determined. According to the energy of each component, four major components are obtained. The main frequencies of these main components are 28.32, 171.90, 314.50 and 398.90 Hz, which are about 1, 6, 11 and 14 times the rotation frequency of the system spindle, respectively. After Hilbert envelope demodulation, it can be seen that the combination of the amplitude modulation signal with a half oscillation period of 0.15D/V_0_(dimension time of 0.21 s, frequency of 4.88 Hz) and the frequency modulation signal with a half oscillation frequency in the mode1 component. As the number of decomposition components increases, in the mode4 component, the main frequency of the component is also increased to 171.90 Hz. In this component, a signal with 11 times the rotation frequency of the system spindle is identified in the pressure signal, and it is modulated by the frequency modulation signal with 3 times the oscillation frequency. Three significant peak frequency signals are obtained. In the mode7 and 8 components, the dominant frequency of separation obtained gradually increases. In addition to having the same frequency modulation characteristic as mode1 and 4, these two components also have the characteristic of beat vibration, which is the sum of two cosine signals with similar frequency appearing in the component with the increase of the number of decomposition. Under the influence of 19.53 Hz = 28.32 Hz-8.79 Hz between the two dominant frequencies of mode1, the typical beat vibration characteristics of 314.5 Hz and 335 5 Hz appear in the component of mode7. Under the influence of the double oscillation frequency of 9.766 Hz, the mode8 component's main frequencies of 398.9 Hz and 409.2 Hz show typical beat vibration characteristics.

These characteristics of the oil pulsating flow pressure signal can be explained as follows. As for the small Reynolds number Re = 138.4 of the experimental device, the oil in the experimental pipe section enters the development section of the experimental pipe section through the sudden expansion region under the pulsation of the rotation frequency of the system's main shaft and an axisymmetric recirculation region is established downstream of the sudden expansion. Oil deviating from its center line causes a strong inflection point in the velocity profile. As the length of the oil entering the test section increased further, the curved streamlines could not remain attached, the flow became unstable, and a vortex structure that led to turbulence was formed. When the fluid in the separation zone is continuously carried into the mainstream by the shear layer, the flow through the vortex rupture immediately restores its stability. The thickness of the recirculation zone decreases as the reattachment point moves downstream. The Komenda effect again deflects the internal asymmetric jet stream (whose asymmetry is maintained by the cross-flow pressure gradient generated in the stable region), and the process repeats indefinitely. Thus, mass-circulation discharge in the recirculation zone leads to continuous stable, unstable, and turbulent flow, followed by a return to a steady-state. This explains the obvious periodicity of the spectrum at the position of pressure signal 2 (x/D = 7) when the Re number of the oil in the experimental device is 138.4. The signal time trajectory clearly shows the periodicity of this phenomenon. This indicates that the oil here alternates between the two velocity levels. The higher velocity is close to V0 horizontally, that is, the average velocity of the section of the test pipe, which corresponds to the center line of the internal jet, indicating that the flow is laminar. Lower velocity levels are associated with high- frequency flow fluctuations. It is shown that the flow velocity is uniform in the profile, and the flatness of the velocity profile is due to the disruption of turbulence. This results in higher pulsation frequency, resulting in flow decomposition and periodic variation of the re-stabilized region. Iribarne et al.^24^ observed that the flow "becomes more and more irregular as it approaches the attachment point" and that the flow acquires significant eddy motion in the recirculation region. This is similar to using time data to reconstruct the three-dimensional velocity field; the unique observations of different shapes of the hairpin structure families obtained from the reconstructed velocity fields are consistent^22^.

## Conclusions

The pressure signal of oil pulsating flow is a kind of multi-component signal; to realise the effective separation of the multi-component signal of pressure, the vibration characteristics are extracted. The abrupt expansion experimental device was described in detail; after the oil at the inlet with an initial velocity of V_0_ = 0.0362 m/s and Re = 138.4 passed through the development section of the pipeline, it flowed into the test section and ran in the experimental pipeline for 10 min. After the change of various indicators of the oil in the system was relatively stable, the data sampling of each parameter was started. According to the pressure sensor 2 installed in the test section, the pressure signal data of the test section was obtained by using LabVIEW. After verifying the central frequency slope criterion to determine the number of components of VMD decomposition, and the method of energy value to determine the main components of the signal, the pressure signal was decomposed and Hilbert envelope demodulation analysis was carried out by using variational mode decomposition.

It is shown that in the absence of finite disturbance, the geometrical complexity of the pipeline leads to adverse pressure gradients and jet deflection closer to the wall, resulting in flow transition, and the beginning of transition is dependent on the location and time of the fluid. Therefore, to obtain the multi-scale and quasi-periodic non-stationary signal characteristics of oil pulsating flow, the center frequency slope criterion is used to determine the number of components of VMD decomposition, and the energy value is used to judge the main components of the signal. The oil pressure fluctuation signal is analysed and processed by VMD. The frequency component information of each major component was obtained, in which the time domain distribution and spectrum of oil pulsating flow pressure signal 2 (at x/D = 7, Re = 138.4) were determined by the slope criterion of the center frequency to determine the number of 9 components decomposed by VMD. According to the energy value of each component, four main components with main frequencies of 28.32, 171.90, 314.50 and 398.90 Hz are obtained. The main frequencies of these main components are about 1, 6, 11 and 14 times of the rotation frequency of the system spindle, respectively. The combination of the amplitude modulation signal with 1/2 oscillation period and the frequency modulation signal with one oscillation frequency in the mode1 component. The main frequency of the component in the mode4 component is 171.90 Hz, which indicates that there is a signal in the pressure signal with a frequency of 11 times the rotation of the system spindle. It is modulated by a frequency modulation signal with a frequency of 3 times the oscillation frequency, and three significant peak frequency signals are obtained. In mode7 and 8 components, in addition to the same frequency modulation characteristics as mode1 and 4, these two components also have the characteristics of beat vibration. Under the influence of 19.53 Hz = 28.32 Hz-8.79 Hz between the two dominant frequencies of mode1, the typical beat vibration characteristics of 314.5 Hz and 335 5 Hz appear in the component of mode7. Under the influence of the double oscillation frequency of 9.766 Hz, the mode8 component's main frequencies of 398.9 Hz and 409.2 Hz show typical beat vibration characteristics.

At this low Reynolds number, the results of steady flow in the experimental segment show that the flow downstream of the contraction section is asymmetrical. The jet from the abrupt expansion deflects toward the wall, resulting in the formation of a unilateral recirculation region. This asymmetry is caused by the Komenda-type wall attachment already observed in a sudden expanding flow in a plane of symmetry. When the oil flows near the pressure sensor 2 (x/D = 7), the separation surface cannot remain attached, and unsteady flow begins. The low-frequency axial oscillation of the reattachment point and the slow, eddy motion of the jet was observed. This phenomenon is associated with the periodic discharge of unstable recirculation zones, resulting in alternating laminar and turbulent flows. The resulting flow is non-stationary and intermittent.

The results show that the proposed center frequency slope criterion method can effectively decompose the pressure signal of oil pulsating flow with VMD, and the correlation between each component and the pressure signal as well as the energy value calculation of each component can obtain the pressure signal with the characteristics of amplitude modulation, frequency modulation and beat vibration.

## Data Availability

The datasets used during the current study are available from the corresponding author on reasonable request.
